# Leveraging Mobile Health to Manage Mental Health/Behavioral Health Disorders: Systematic Literature Review

**DOI:** 10.2196/42301

**Published:** 2022-12-27

**Authors:** Clemens Scott Kruse, Jose A Betancourt, Matthew Gonzales, Kennedy Dickerson, Miah Neer

**Affiliations:** 1 School of Health Administration Texas State University San Marcos, TX United States

**Keywords:** mHealth, telemedicine, mental health, behavioral health, anxiety, mobile device, smartphone, SMS text messaging, RCT

## Abstract

**Background:**

Mental health is a complex condition, highly related to emotion. The COVID-19 pandemic caused a significant spike in depression (from isolation) and anxiety (event related). Mobile Health (mHealth) and telemedicine offer solutions to augment patient care, provide education, improve symptoms of depression, and assuage fears and anxiety.

**Objective:**

This review aims to assess the effectiveness of mHealth to provide mental health care by analyzing articles published in the last year in peer-reviewed, academic journals using strong methodology (randomized controlled trial).

**Methods:**

We queried 4 databases (PubMed, CINAHL [Cumulative Index to Nursing and Allied Health Literature], Web of Science, and ScienceDirect) using a standard Boolean search string. We conducted this systematic literature review in accordance with the Kruse protocol and reported it in accordance with the PRISMA (Preferred Reporting Items for Systematic Reviews and Meta-Analysis) 2020 checklist (n=33).

**Results:**

A total of 4 interventions (mostly mHealth) from 14 countries identified improvements in primary outcomes of depression and anxiety as well as in several secondary outcomes, namely, quality of life, mental well-being, cognitive flexibility, distress, sleep, self-efficacy, anger, decision conflict, decision regret, digestive disturbance, pain, and medication adherence.

**Conclusions:**

mHealth interventions can provide education, treatment augmentation, and serve as the primary modality in mental health care. The mHealth modality should be carefully considered when evaluating modes of care.

**Trial Registration:**

PROSPERO International Prospective Register of Systematic Reviews CRD42022343489; https://www.crd.york.ac.uk/PROSPERO/display_record.php?RecordID=343489

## Introduction

### Rationale

Mental health is a complex topic that is highly related to emotion, because emotional regulation is necessary for daily functioning [1]. Emotional regulation is necessary for friendships and intimate relationships [2]. The perception of mental health can be culturally based and highly related to self-actualization [3]. For the purposes of this manuscript, we define mental health as being able to work creatively and productively, to relate to others in a way that is mutually satisfying, and to feel comfortable when alone, usually developing a rich and fulfilling inner life [4]. The psychopathology of mental illness is also complicated. Mental illness can manifest itself in terms of depression and anxiety, which are the major foci of this manuscript. Depression can be described as a mood (sadness or lack of enjoyment), a symptom, a syndrome, or a disorder [5]. During the COVID-19 pandemic, college students were particularly affected by depression due to the isolation created by the pandemic thrust upon them during a time in their lives when they expected to be highly socially active [6]. Anxiety often refers to multiple mental and physiological phenomena, including fear, distress, and a constant state of worry over events or actual situations [7]. The pandemic created COVID-19 anxiety, which has been added to a list of anxiety disorders [8].

Telemedicine and telehealth are defined as healing at a distance using information communication technologies to improve health outcomes [9]. The World Health Organization does not distinguish between these terms, so they will be used interchangeably in this manuscript. Telemedicine has existed for decades, but it became an essential modality of care during the COVID-19 pandemic. The highly contagious nature of COVID-19 prevented face-to-face appointments, and many providers were forced into this modality before they thought they were ready [10]. Despite this challenge, many providers discovered the effectiveness this modality can enable, including the use of mobile health (mHealth) and eHealth [11]. mHealth is a component of eHealth and telehealth that enables medical and public health practices through mobile devices, such as smartphones, patient monitoring devices, and other wireless devices [12]. Mobile devices have blurred the lines between tablets and computers, and the computing power of mobile devices enables the use of many apps formerly only available on a computer. This study focuses on the intersection between mHealth and mental health care.

Studies have shown that some college students are comfortable with mental health screening through mHealth modalities in the areas of performance expectancy and social influence [13]. Social influence and the stigma associated with mental health care greatly influence this age bracket (18-24 years) and their willingness to answer questions over their mobile devices. Telemedicine offers safety and efficiency, but several limitations prevent wide adoption such as technical problems, patient distraction, lack of confidentiality, compromised therapeutic alliance, and the management of unstable patients through a distance modality [6].

mHealth for an older population has not been entirely successful as well mostly due to the digital divide inherent to older populations and overall digital literacy [14]. Practitioners recognize this gap, and some have even tried to bridge the gap with intensive training to enable their patients to participate in this modality of care. Unfortunately, they found that a substantial number of older adults either do not have the technology or cannot negotiate the technology, despite this training. This population experiences high rates of depression, either due to illnesses, such as cancer (incidence as high as 58%) [15], or dementia (incidence as high as 34%) [16]. The overall prevalence of depression among older adults is estimated at 28.4% [17].

One systematic review from 2022 analyzed 26 articles over 10 years [18]. It found a major reduction in symptoms of depression among trials that included participants with moderate to severe depression, but not as strong as an effect among trials involving participants with mild to moderate depression. The reviewers concluded that app-based interventions have a moderate effect to reduce symptoms of depression.

Another systematic review from 2022 analyzed 21 articles about telepsychiatry over 15 years [19]. It identified familiar themes such as equivalence to in-person, convenience, overcoming remoteness, and timely access to treatment with specialists. The reviewers concluded that the video teleconferencing technology enables telepsychiatry in both the home and the emergency department on par with in-person reviews with the additional benefit of wider access and timeliness to treatment.

### Objectives

The purpose of this review is to analyze studies published over the last year that examine mHealth as an intervention to both screen for and treat symptoms of mental health among adults aged over 18 years with strong methodological design (randomized controlled trial [RCT]). The intention of this review is to focus on mental health interventions during 1 year of the COVID-19 pandemic.

## Methods

### Protocol and Registration

This review is conducted in accordance with the Kruse protocol for writing a systematic review. It is reported in accordance with the PRISMA (Preferred Reporting Items for Systematic Reviews and Meta-Analysis) checklist. This review is registered with PROSPERO (registration number CRD42022343489).

### Eligibility Criteria

This review focused on studies with strong methodology including participants who were adults (>18 years of age) that were published in peer-reviewed, academic journals over the last year. Other systematic reviews were excluded to avoid confounding the results. All reporting is in accordance with the PRISMA 2020 standard [20].

### Information Sources

A total of 4 research databases were queried: PubMed (MEDLINE), CINAHL (Cumulative Index to Nursing and Allied Health Literature), Web of Science, and ScienceDirect from Embase. They were queried on August 14, 2022. These databases were chosen due to their availability and comprehensive indexing of research. Their availability make it easier for others to duplicate our study. Their comprehensive indexing ensures we capture a majority of the literature in our search.

### Search Strategy

Our initial process included a search on Google Scholar to better understand the topic and recent work published. We gathered information from Google Scholar into an MS Excel spreadsheet to enable the review team to read the articles so that we could see the key terms these studies used for indexing. Using the Medical Subject Headings (MeSH) from the US National Library of Medicine, we created a Boolean search string to exhaustively query the databases without redundant terms. We used the same search strategy in all databases and similar filter strategies.

### Selection Process

In accordance with the Kruse protocol [21], we searched key terms in all databases using a Boolean search string: (mhealth OR “mobile app” OR telemedicine) AND (“mental health” OR “behavioral health” OR “depression”). We removed duplicates, filtered the results, and screened abstracts for applicability to our objective statement [21]. We selected only studies with strong methods (eg, RCT). The RCT and true experiments were chosen due to their rigorous adherence to control groups and comparisons to the same.

### Data Collection Process

The Kruse protocol standardized an MS Excel spreadsheet as a data extraction tool and as an analysis tool [21]. The spreadsheet’s standardized fields allowed the collection of additional data at each step of the process, thus making analysis robust and useful to both clinicians and administrators. Through 3 consensus meetings we finalized the group of articles for analysis, identified themes, and finalized the additional analysis through a data synthesis method.

### Data Items

In accordance with the Kruse protocol [21], we collected the following fields of data at each step: Google Scholar step (date of publication, authors, study title, journal, impact factor from Journal Citations Reports, study design, key terms, experimental intervention, results, and comments from each reviewer); filter articles step (the number of results before and after each filter was applied in all 4 databases); abstract screening step (database source, date of publication, authors, study title, journal, screening decision for each reviewer, notes about rejections, consensus meeting 1 determination of screening decision, and a set of rejection criteria); analysis step (database source, date of publication, authors, study title, participants, experimental intervention, results compared with a control group, medical outcomes, study design, sample size, bias effect size, country of origin, statistics used, patient satisfaction, facilitators to adoption, barriers to adoption, and the strength and quality of evidence).

### Study Risk and Reporting of Bias Assessment

Risk of bias was determined through multiple means. Reviewers used the John’s Hopkins Nursing Evidence-Based Practice (JHNEBP) tool assessment of the strength and quality of evidence [22]. Strength of evidence is based on the study design (eg, RCT, quasi-experimental, qualitative), and the quality of evidence is based on adequate sample size, adequate control, and consistency of results. Reviewers also made a note of other observations of bias such as selection, sample, design, or publication bias. These observations and the JHNEBP assessment of the strength and quality of evidence were used for interpreting the results, because bias can limit the internal and external validity of the results [23].

### Effect Measures

The preferred methodologies for this review were the RCT and true experiments because these are the strongest group of methodologies in the JHNEBP tool. The preferred measures of effect were the Cohen *d*, but other measures of effect were also collected, such as the odds ratio. All measures of effect were tabulated for those studies in which they were reported.

### Synthesis Methods

We performed a thematic analysis of the data extracted [24]. This thematic analysis helped us make sense of the data by grouping same or similar observations into themes. Themes and individual observations were then tabulated for both reporting and to enable inferences.

### Additional Analyses and Certainty Assessment

Sensitivity, specificity, and effect size were tabulated and included in the data extraction. Combined with the narrative analysis, this provided us with certainty assessments. The frequency of themes does not imply importance, but it does provide confidence in the data analyzed.

### Ethics Approval

No human subjects were used in this research. It is therefore IRB exempt.

## Results

### Overview

**Figure 1:**
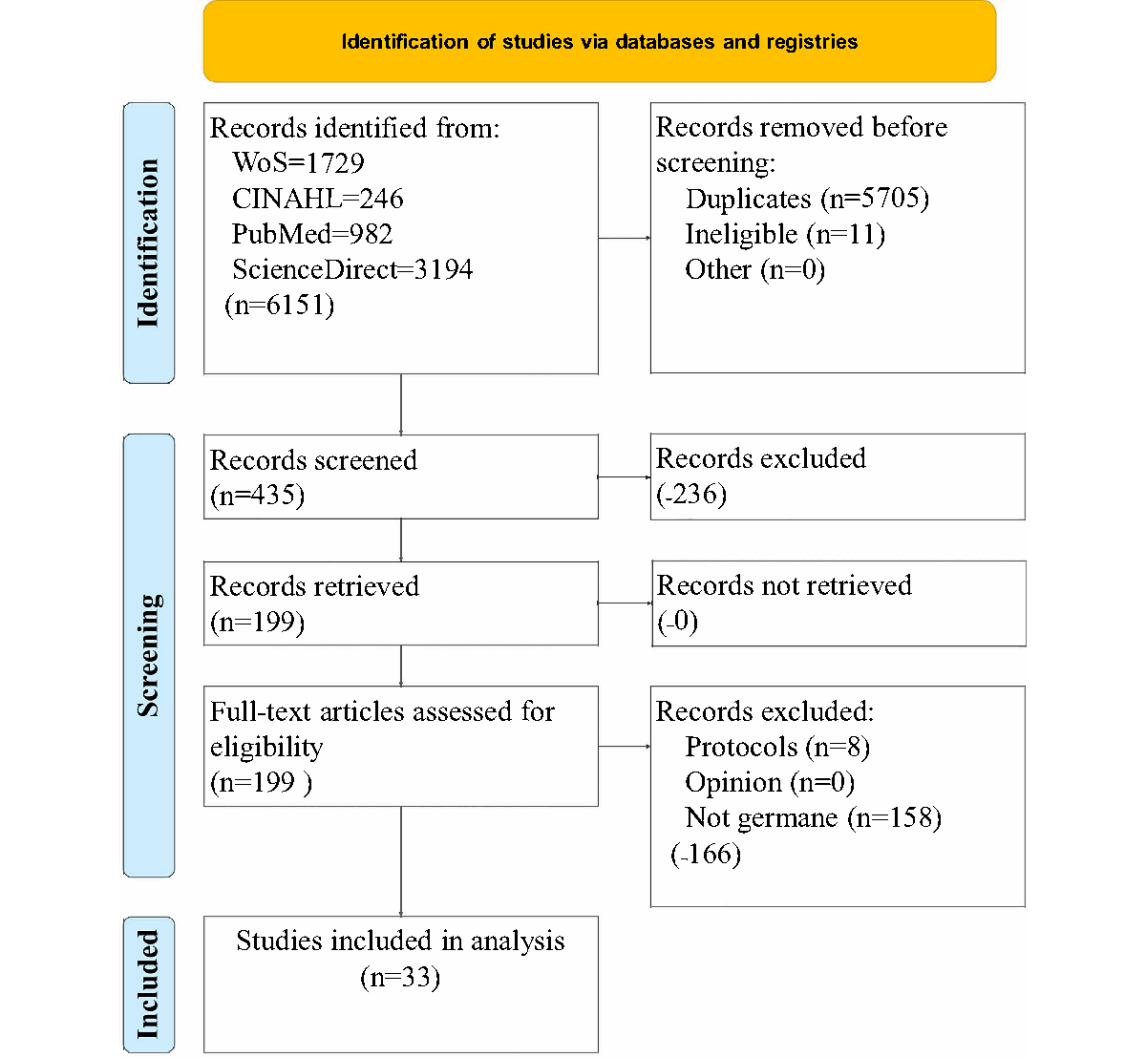
Article selection process. CINAHL: Cumulative Index to Nursing and Allied Health Literature; WoS: Web of Science.

Figure 1 illustrates the study selection process. The query of 4 databases resulted in 23,713 results; however, 21,426 of these results were duplicates. After filtering and screening, reviewers were left with 67 articles eligible for review. The reviewers chose to only analyze the RCTs, because this is the highest strength of evidence in the JHNEBP. This can be seen in Figure 1 under “methods not strong,” meaning we excluded all but RCTs and true experiments. The remaining group of studies for analysis was 33.

### Study Selection

#### Study Characteristics

Following the PRISMA checklist [20] and the Kruse protocol [21], we extracted the following data fields: participants, intervention, comparison with the control or other group, medical outcomes, and study design (PICOS; Table 1) as a way to summarize study characteristics, as required by PRISMA. Of the 33 studies analyzed over the 1-year period, 17 were from 2021 [25-41] and 16 were from 2022 [42-57]. All studies were RCTs involving adults, and 48% (16/33) involved an average age of 50 years or above. About 64% (21/33) used an mHealth or eHealth app as the intervention, 21% (7/33) used telemedicine over computer or mobile device, 12% (4/33) used telephone, and 3% (1/33) used telemonitoring. Studies originated from 14 countries, but 42% (14/33) originated from the United States and 9% (3/33) from China, which accounted for over half of the studies.

**Table 1:** Summary of study characteristics (PICOS^a^).

Authors	Participants	Experimental intervention	Results (compared with the control group)	Medical outcomes reported	Study design
Acierno et al [25]	Adult females with PTSD^b^ from sexual trauma, average age 43.4 years, 64% African American	Telemedicine	Reduced depression, but there were no differences in dose received or PTSD symptom reduction	Decrease in depression, but not statistically significant	RCT^c^
Baek et al [26]	Adults, average age 41.9 years, 67% female	mHealth^d^ app (MibyeongBogam [MBBG])	The intervention group showed a decrease in depression (P=.003), anxiety (P=.01), sleep disturbance (P=.02), anger (P=.003), and pain (P=.02) greater than the control group; also fatigue (P=.6) and digestive disturbance (P=.76) were not statistically significant	Decreased depression, anxiety, sleep disturbance, anger, pain, fatigue, digestive disturbance	RCT
Colomina et al [27]	Adults, average age 73 years, 66% female	mHealth self-management app (CONNECARE)	Decreased anxiety more than the control group, but there were no differences in depression symptom reduction	Decreased anxiety, but not statistically significant over traditional care	RCT
Dobkin et al [28]	Adult veterans, average age 67.8 years, 100% male, 92% White	Video-to-home cognitive behavioral therapy	Intervention outperformed treatment as usual across all 3 measures of depression (P=.001), decreased anxiety, but not statistically significant	Decreased depression and anxiety	RCT
Domogalla et al [29]	Adults with psoriasis, average age 49 years, 60% male	mHealth study and disease management app	Significant reduction in HADS^e^, HADS-D^f^ (P=.04), and HADS-A^g^ (P=.05) more than those in the control group	Decreased anxiety and depression	RCT
Fang et al [30]	Adults 100% female	mHealth app (Pink Journey)	Decreased anxiety, depression, decision conflict, and decision regret more than the control group, but not statistically significant from control; decreased body image distress (P=.027)	Decreased anxiety, depression, decision conflict, decision regret, and body image distress	RCT
Fortney et al [31]	Adults average age 39 years, 70% female, 66% White	Telepsychiatry	Decreased depression and anxiety, but with small effect	Decreased depression and anxiety	RCT
Huberty et al [32]	Adults average age 44.2 years, 78% female, 56% White	mHealth app	Decreased anxiety (P<.001) and depression (P<.001) more than the control group	Decreased anxiety and depression	RCT
Jones et al [33]	Adults average age 53.4 years	mHealth app (WRAP)	Decreased HADS more than the control group	Decreased anxiety and depression	RCT
Krzyzanowska et al [34]	Adults <40<75 years, median age 55 years	Telephone	No effect on anxiety, depression, or self-efficacy	No effect on anxiety, depression, or self-efficacy	RCT
Moskowitz et al [35]	Adults average age 37.95 years, 74% female	eHealth	Decreased depression (P<.06) more than the control group	Decreased depression	RCT
Pakrad et al [36]	Adults average age 62.7 years, 82% male	mHealth app	Decreased anxiety (P<.028), stress (P<.022), and quality of life (P<.001) more than the control. Decreased depression more than the control group, but not statistically significant (P<.063)	Decreased depression, anxiety, and stress, and increased quality of life	RCT
Rollman et al [37]	Adults average age 63.9 years, 56% male, 74% White	Telephone	Decreased depression more than the control group	Decreased depression	RCT
Romijn et al [38]	Adults, average age 36.25 years, 75% White	eHealth cognitive behavioral therapy (inference-based cognitive behavioral therapy)	Decreased anxiety more than the control group	Decreased anxiety	RCT
Su and Yu [39]	Adults, average age 55.75 years, 85% male, 100% Chinese	eHealth	Decreased anxiety more than the control group, no effect on depression	Decreased anxiety	RCT
Taguchi et al [40]	Adults, average age 50 years, 67% female	Video-based cognitive behavioral therapy	Decreased depression and anxiety (not statistically significant from the control)	Decreased depression and anxiety	RCT
Wong et al [41]	Adults >60 years, average age 72 years, 82% female	mHealth app	Decreased depression (not statistically significant over the control group), increased medication adherence (P<.001), self-efficacy (P<.16), and quality of life (P<.04)	Decreased depression, improved medication adherence, self-efficacy, and quality of life	RCT
Aikens et al [42]	Adults, average age 48.6 years, 81% female, 74% White	Telephone (automated interactive voice response)	Decreased depression more than the control group, with medium effect; increased self-efficacy	Decreased depression, increased self-efficacy	RCT
Akin-Sari et al [43]	Adults, average age 23 years, 78% female	mHealth app	Decreased depression and COVID-19 distress more than the control group	Decreased depression, decreased COVID-19 distress	RCT
Bathgate et al [44]	Adults, average age 32.4 years, 81% female, 97% White	Telemedicine	Decreased depression more than the control (P=.78), anxiety but not more than the control (P=.6), increased coping self-efficacy but not more than the control (P=.93), increased quality of life (physical functioning, social functioning, and vitality)	Decreased depression and anxiety, increased coping self-efficacy and quality of life	RCT
Catuara-Solarz et al [45]	Adults, average age 40 years, 54% female	mHealth app	Decreased anxiety (P=.04), increase in resilience (P=.001), sleep (P=.01), and mental well-being (P=.02) more than the control group	Decreased anxiety, increase in resilience, sleep, and mental well-being	RCT
Deady et al [46]	Adults, average age 40 years, 74% male	mHealth app (HeadGear)	Improved depression, anxiety, resilience, and well-being more than the control group (P=.0031)	Improved depression, anxiety, resilience, and well-being	RCT
Drew et al [47]	Adults, average age 48.4 years, 100% male	eHealth app (SHED-IT)	Improved depression, sleep, cognitive flexibility more than the control	Improved depression, sleep, cognitive flexibility	RCT
Guo et al [48]	Adults, average age 28.3 years, 95% male, 100% Chinese	mHealth, social media (Run4Love)	Improved depression more than the control	Decreased depression	RCT
Gustafson et al [49]	Adults, >65 years, average age 76.5 years, 74% female, 89% White	eHealth app (ElderTree)	Improved depression (OR^h^ –0.20, P=.034) and overall mental health quality of life (OR 0.32, P=.007) more than the control group	Decreased depression, increased mental health, increased quality of life	RCT
Kuhn et al [50]	Adults, average age 44.5 years, mostly male	mHealth app	Decreased depression (*d*=–0.8, P<.012) and sleep-related impairment (*d*=–0.6, P<.04) more than the control group	Decreased depression and sleep-related impairment	RCT
Lopez et al [51]	Adults, average age 44 years, 100% female	Telemedicine	Reduced depression, but there were no differences in dose received or PTSD symptom reduction	Decrease in depression, but not statistically significant	RCT
Mitchell et al [52]	Adults, average age 51 years, 60% female	Telemedicine cognitive behavioral therapy (RED-D)	Decreased depression and readmission (P<.012) more than the control	Decreased depression	RCT
Nardi et al [53]	Adults, average age 42.9 years, 93% female	mHealth app (unwinding anxiety)	Decreased anxiety (P=.005) and worry (P=.01) more than the control	Decreased anxiety and worry	RCT
Orman et al [54]	Adults, average age 68.4 years, 64% male	Telephone	Decreased depression and anxiety greater than usual care, short-term positive effect on quality of life	Decreased anxiety and depression, and increased quality of life	RCT
Sun et al [55]	Adults, 100% Chinese	mHealth app (mindfulness)	Decreased depression and anxiety (P=.024) greater than usual care, but depression was not statistically different	Decreased anxiety and depression	RCT
Volpato et al [56]	Adults, average age 76.2 years, 51% male	mHealth cognitive behavioral therapy	Decreased anxiety and depression, but not statistically significant than the control. Improved adherence to noninvasive ventilation (P<.001) and quality of life (P<.002)	Decreased anxiety and depression, improved quality of life, and noninvasive ventilation	RCT
Ware et al [57]	Adults, average age 59 years, 56% male	Telemonitoring	No effect on anxiety or depression. Improved self-care maintenance, management, confidence, and physical quality of life	Improved self-care maintenance, management, confidence, and physical quality of life	RCT

^a^PICOS: participants, intervention, comparison with the control or other group, medical outcomes, and study design.

^b^PTSD: posttraumatic stress disorder.

^c^RCT: randomized controlled trial.

^d^mHealth: mobile health.

^e^HADS: Hospital Anxiety and Depression Scale

^f^HADS-D: Hospital Anxiety and Depression Scale-Depression.

^g^HADS-A: Hospital Anxiety and Depression Scale-Anxiety.

^h^OR: odds ratio.

#### Risk of Bias in and Across Studies

The JHNEBP quality assessment tool identified the strength and quality of evidence [22]. Strength of evidence is defined by methodology: level I is defined as true experiments and RCTs; level II is defined as quasi-experiments; and level III is defined as nonexperimental, observational, and qualitative studies. Levels IV and V are defined as expert opinions and editorials. We only used RCTs in our systematic literature review, so the group for analysis was 100% (33/33) level I. The quality of evidence in the JHNEBP tool is defined by sample size, consistency of results (based on established measurement standards), control groups, conclusions, and literature reviews. Each level accepts a lower standard. Level A is defined by consistent results with sufficient sample sizes (based on power analysis), adequate control groups, definitive conclusions, and consistent recommendations based on extensive literature reviews. Level B is defined by reasonably consistent results, sufficient sample sizes, some control groups, fairly definitive conclusions, and reasonably consistent recommendations based on fairly comprehensive literature reviews. Level C is defined by little evidence with inconsistent results, insufficient sample sizes, and nondefinitive conclusions. In our group for analysis, only 1 RCT (1/33, 3%) was defined as level B, while the rest were defined as level A (32/33, 97%).

Reviewers also noted instances of bias, because bias can limit external and internal validity [23]. There were 30 instances of selection bias (affecting internal validity), and 28 instances of sample bias (affecting external validity). The latter were usually due to a high percentage of sex or race in the sample. The former were due to studies conducted in 1 region of 1 country.

#### Results of Individual Studies

Table 2 summarizes the results of individual studies through themes. Themes were identified when the same or similar observation occurred in the literature. An observation-to-theme match can be found in Multimedia Appendices 1 and 2. The other data items collected (sample size, bias, effect size, country of origin, statistics used, and JHNEBP strength and quality of evidence ratings) can be found in Multimedia Appendix 3. The average sample size was 331.

**Table 2:** Summary of themes, sorted chronologically by author.

Authors	Intervention themes	Results themes	Medical outcomes themes	Patient satisfaction themes	Effectiveness themes	Barrier themes
Acierno et al [25]	Telemedicine	Reduced depression No statistical significance for at least one condition	Reduced depression	Satisfied	Reduced depression Enabled preference for telemedicine	May not be the preferred treatment method Staff training Low reimbursement
Baek et al [26]	mHealth^a^/eHealth app	Reduced depression Reduced anxiety Increased sleep Decreased anger Decreased pain Decreased digestive disturbance No statistical significance for at least one condition	Reduced depression Reduced anxiety Increased sleep Decreased anger Decreased pain Decreased digestive disturbance	Not reported	Reduced depression Reduced anxiety Increased sleep Decreased anger Decreased pain Decreased digestive disturbance	May not be the preferred treatment method Staff training Low reimbursement
Colomina et al [27]	mHealth/eHealth app	Reduced anxiety No effect on depression No statistical significance for at least one condition	Reduced anxiety	Satisfied	Reduced health costs per patient Reduced anxiety	May not be the preferred treatment method Staff training Low reimbursement
Dobkin et al [28]	Telemedicine	Reduced depression Reduced anxiety No statistical significance for at least one condition	Reduced anxiety Reduced depression	Satisfied	Reduced anxiety Reduced depression Extended care to rural patients	May not be the preferred treatment method Staff training Low reimbursement
Domogalla et al [29]	mHealth/eHealth app	Reduced anxiety Reduced depression	Reduced anxiety Reduced depression	Satisfied	Reduced anxiety Reduced depression	May not be the preferred treatment method Staff training
Fang et al [30]	mHealth/eHealth app	Reduced anxiety Reduced depression Decreased decision conflict Decreased decision regret Decreased distress No statistical significance for at least one condition	Reduced anxiety Reduced depression Decreased decision conflict Decreased decision regret Decreased distress	Satisfied	Reduced anxiety Reduced depression Decreased decision conflict Decreased decision regret Decreased distress	May not be the preferred treatment method Staff training Low reimbursement
Fortney et al [31]	Telemedicine	Reduced anxiety Reduced depression	Reduced anxiety Reduced depression	Not reported	Reduced anxiety Reduced depression	May not be the preferred treatment method Staff training Low reimbursement
Huberty et al [32]	mHealth/eHealth app	Reduced anxiety Reduced depression	Reduced anxiety Reduced depression	Satisfied	Reduced anxiety Reduced depression	May not be the preferred treatment method Staff training
Jones et al [33]	mHealth/eHealth app	Reduced anxiety Reduced depression	Reduced anxiety Reduced depression	Satisfied	Reduced anxiety Reduced depression	May not be the preferred treatment method Staff training
Krzyzanowska et al [34]	Telephone	No effect on anxiety No effect on depression No effect on self-efficacy	None	Not reported	None	May not be the preferred treatment method Staff training
Moskowitz et al [35]	mHealth/eHealth app	Reduced depression	Reduced depression	Satisfied	Reduced depression	May not be the preferred treatment method Staff training Low reimbursement
Pakrad et al [36]	mHealth/eHealth app	Reduced anxiety Decreased distress Increased quality of life Reduced depression No statistical significance for at least one condition	Reduced anxiety Decreased distress Increased quality of life Reduced depression	Satisfied	Reduced anxiety Decreased distress Reduced depression Increased quality of life	May not be the preferred treatment method Staff training Low reimbursement
Rollman et al [37]	Telephone	Reduced depression	Reduced depression	Not reported	Reduced depression	May not be the preferred treatment method Staff training Low reimbursement
Romijn et al [38]	mHealth/eHealth app	Reduced anxiety	Reduced anxiety	Satisfied	Reduced anxiety	May not be the preferred treatment method Staff training
Su and Yu [39]	mHealth/eHealth app	Reduced anxiety No effect on depression	Reduced anxiety	Not reported	Reduced anxiety	May not be the preferred treatment method Staff training
Taguchi et al [40]	Telemedicine	Reduced anxiety Reduced depression No statistical significance for at least one condition	Reduced anxiety Reduced depression	Not reported	Reduced anxiety Reduced depression	May not be the preferred treatment method Staff training
Wong et al [41]	mHealth/eHealth app	Reduced depression No statistical significance for at least one condition Increased medication adherence Increased self-efficacy Increased quality of life	Reduced depression Increased medication adherence Increased self-efficacy Increased quality of life	Not reported	Reduced depression Increased medication adherence Increased self-efficacy Increased quality of life	May not be the preferred treatment method Staff training Low reimbursement
Aikens et al [42]	Telephone	Reduced depression Increased self-efficacy	Reduced depression Increased self-efficacy	Not reported	Reduced depression Increased self-efficacy	May not be the preferred treatment method Staff training Low reimbursement
Akin-Sari et al [43]	mHealth/eHealth app	Reduced depression Decreased distress	Reduced depression Decreased distress	Not reported	Reduced depression Decreased distress	May not be the preferred treatment method Staff training Low reimbursement
Bathgate et al [44]	Telemedicine	Reduced depression Reduced anxiety Increased self-efficacy Increased quality of life No statistical significance for at least one condition	Reduced depression Reduced anxiety Increased self-efficacy Increased quality of life	Satisfied	Reduced depression Reduced anxiety Increased self-efficacy Increased quality of life	May not be the preferred treatment method Staff training Low reimbursement
Catuara-Solarz et al [45]	mHealth/eHealth app	Reduced anxiety Decreased fatigue/increased resilience Increased sleep Increased mental well-being/cognitive flexibility	Reduced anxiety Decreased fatigue/increased resilience Increased sleep Increased mental well-being/cognitive flexibility	Satisfied	Reduced anxiety Decreased fatigue/increased resilience Increased sleep Increased mental well-being/cognitive flexibility	May not be the preferred treatment method Staff training
Deady et al [46]	mHealth/eHealth app	Reduced depression Reduced anxiety Decreased fatigue/increased resilience Increased mental well-being/cognitive flexibility	Reduced depression Reduced anxiety Decreased fatigue/increased resilience Increased mental well-being/cognitive flexibility	Not reported	Reduced depression Reduced anxiety Decreased fatigue/increased resilience Increased mental well-being/cognitive flexibility	May not be the preferred treatment method Staff training
Drew et al [47]	mHealth/eHealth app	Reduced depression Increased sleep Increased mental well-being/cognitive flexibility	Reduced depression Increased sleep Increased mental well-being/cognitive flexibility	Not reported	Reduced depression Increased sleep Increased mental well-being/cognitive flexibility	May not be the preferred treatment method Staff training
Guo et al [48]	mHealth/eHealth app	Reduced depression	Reduced depression	Not reported	Reduced depression	May not be the preferred treatment method Staff training
Gustafson et al [49]	mHealth/eHealth app	Reduced depression Increased mental well-being/cognitive flexibility Increased quality of life	Reduced depression Increased mental well-being/cognitive flexibility Increased quality of life	Not reported	Reduced depression Increased mental well-being/cognitive flexibility Increased quality of life	May not be the preferred treatment method Staff training Low reimbursement
Kuhn et al [50]	mHealth/eHealth app	Reduced depression Increased sleep	Reduced depression Increased sleep	Not reported	Reduced depression Increased sleep	May not be the preferred treatment method Staff training Low reimbursement
Lopez et al [51]	Telemedicine	Reduced depression No statistical significance for at least one condition	Reduced depression	Not reported	Reduced depression	May not be the preferred treatment method Staff training Low reimbursement
Mitchell et al [52]	Telemedicine	Reduced depression	Reduced depression	Satisfied	Reduced depression Decreased readmissions	May not be the preferred treatment method Staff training Low reimbursement
Nardi et al [53]	mHealth/eHealth app	Reduced anxiety Decreased distress	Reduced anxiety Decreased distress	Not reported	Reduced anxiety Decreased distress	May not be the preferred treatment method Staff training Low reimbursement
Orman et al [54]	Telephone	Reduced depression Reduced anxiety Increased quality of life	Reduced depression Reduced anxiety Increased quality of life	Not reported	Reduced depression Reduced anxiety Increased quality of life	May not be the preferred treatment method Staff training
Sun et al [55]	mHealth/eHealth app	Reduced depression Reduced anxiety No statistical significance for at least one condition	Reduced depression Reduced anxiety	Not reported	Reduced depression Reduced anxiety	May not be the preferred treatment method Staff training
Volpato et al [56]	mHealth/eHealth app	Reduced anxiety Reduced depression Increased quality of life	Reduced anxiety Reduced depression Increased quality of life	Not reported	Reduced anxiety Reduced depression Increased quality of life	May not be the preferred treatment method Staff training Low reimbursement
Ware et al [57]	Telemonitoring	No effect on anxiety No effect on depression Increased self-efficacy Increased mental well-being/cognitive flexibility Increased quality of life	No effect on anxiety No effect on depression Increased self-efficacy Increased mental well-being/cognitive flexibility Increased quality of life	Not reported	Increased self-efficacy Increased mental well-being/cognitive flexibility Increased quality of life	May not be the preferred treatment method Staff training

^a^mHealth: mobile health.

#### Results of Syntheses, Additional Analysis, and Certainty of Evidence

A thematic analysis helped makes sense of the data collected. Although thematic analyses are typically used for qualitative analysis, other systematic reviews have also used this technique to makes sense of all observations from the data extraction process, whether the studies were qualitative or quantitative [10,11]. The themes and observations are tabulated into affinity matrices for interpretation.

### Patient Satisfaction

Patient satisfaction was not reported in all studies (20/33, 61%); however, 13/33 (39%) reported users were satisfied or highly satisfied. At the point where these data were collected, users were very pleased with the user interface and any further progress in the interventions improved their mental health.

### Results of Interventions, Compared With the Control Groups

Table 3 summarizes the results of interventions compared with the control groups (treatment as usual, in-person). This section is designed for the scientist or researcher. A total of 11 themes and 2 individual observations were identified by the reviewers for a total of 48 occurrences in the literature, whereas 11 themes and 7 observations were noted for a total of 95 observations. In the 33 studies analyzed, 26 (79%) showed an improvement in symptoms of depression [25,26,28-33,35-37,40-44,46-52,54-56], while 19 (58%) showed an improvement in anxiety [26-33,36,38-40,44-46,53-56]. Only 11/33 (33%) reported that at least one symptom was not statistically significant when compared with results from the control group, but the improvement was still noted [25-28,30,36,40,41,44,51,55]. As many as 7/33 (21%) showed an increase in quality of life [36,41,44,49,54,56,57], and 5 (15%) showed an increase in mental well-being, cognitive flexibility, or confidence [45-47,49,57]. A total of 4 themes emerged that each appeared 4/33 times (12%): decreased distress (body image or COVID-19 distress) [30,36,43,53], increased sleep (less sleep disturbance or sleep-related impairment) [26,45,47,50], increased self-efficacy [41,42,44,57], and the intervention had no effect on depression [27,34,39,57]. A total of 2 themes each appeared 2/33 times (6%): Decreased fatigue (increased resilience) [45,46] and the intervention had no effect on anxiety [34,57]. The following observations only occurred once in the literature: decrease in anger [26], decrease in decision conflict [30], decrease in decision regret [30], decreased digestive disturbance [26], decreased pain [26], increased medication adherence [41], and the intervention had no effect on self-efficacy [34].

**Table 3:** Results compared with the control groups.

Results themes and observations	Frequency, n (n=95)
Reduced depression [25,26,28-33,35-37,40-44,46-52,54-56]	26
Reduced anxiety [26-33,36,38-40,44-46,53-56]	19
No statistical significance for at least one condition [25-28,30,36,40,41,44,51,55]	11
Increased quality of life [36,41,44,49,54,56,57]	7
Increased mental well-being/cognitive flexibility [45-47,49,57]	5
Decreased distress [30,36,43,53]	4
Increased sleep [26,45,47,50]	4
Increased self-efficacy [41,42,44,57]	4
No effect on depression [27,34,39,57]	4
Decreased fatigue/increased resilience [45,46]	2
No effect on anxiety [34,57]	2
Decreased anger [26]	1
Decreased decision conflict [30]	1
Decreased decision regret [30]	1
Decreased digestive disturbance [26]	1
Decreased pain [26]	1
Increased medication adherence [41]	1
No effect on self-efficacy [34]	1

### Medical Outcomes Commensurate With the Use of mHealth

Table 4 summarizes the medical outcomes observed. A total of 8 themes and 6 individual observations were recorded commensurate with the adoption of mHealth for the management of mental health for a total of 78 occurrences. The results (compared with the control groups) and medical outcomes were very similar, but they are focused on observations for the provider. The only difference was 1 study [34] reported no effect on depression or anxiety.

**Table 4:** Medical outcomes commensurate with the adoption of mobile health.

Medical outcomes themes and observations	Frequency, n (n=78)
Reduced depression [25,26,28-33,35-37,40-44,46-52,54-56]	26
Reduced anxiety [26-33,36,38-40,44-46,53-56]	19
Increased quality of life [36,41,44,49,54,56,57]	7
Increased mental well-being/cognitive flexibility [45-47,49,57]	5
Decreased distress [30,36,43,53]	4
Increased sleep [26,45,47,50]	4
Increased self-efficacy [41,42,44,57]	4
Decreased fatigue/increased resilience [45,46]	2
Decreased anger [26]	1
Decreased decision conflict [30]	1
Decreased decision regret [30]	1
Decreased digestive disturbance [26]	1
Decreased pain [26]	1
Increased medication adherence [41]	1
None [34]	1

### Effectiveness of mHealth to Manage Mental Health

Table 5 summarizes the themes and observations related to the effectiveness of mHealth in managing mental health. These were highly similar to the results and medical outcomes, but they include observations for the health care administrator. A total of 8 themes and 11 observations were noted for 82 observations. Because of the similarities with the previous tables, only the differences will be reported. One study noted the reduction in readmission when mHealth was used as part of the follow-up to focus on mental health conditions [52]. One study highlighted that while the intervention did not result in a statistically significant reduction in depression and anxiety over normal care, it enabled a preference for telemedicine, if a patient prefers it [25]. One study highlighted how the intervention can extend care to rural patients [28]. Another study highlighted a reduction in cost of care per patient when using mHealth over traditional care [27]. Two studies reported no effects on mental health from the intervention [34,57].

**Table 5:** Clinical and administrative effectiveness of mobile health to manage mental health.

Effectiveness themes and observations	Frequency, n (n=165)
Reduced depression [25,26,28-33,35-37,40-44,46-52,54-56]	26
Reduced anxiety [26-33,36,38-40,44-46,53-56]	19
Increased quality of life [36,41,44,49,54,56,57]	7
Increased mental well-being/cognitive flexibility [45-47,49,57]	5
Decreased distress [30,36,43,53]	4
Increased self-efficacy [41,42,44,57]	4
Increased sleep [26,45,47,50]	4
Decreased fatigue/increased resilience [45,46]	2
Decreased anger [26]	1
None [34,57]	2
Decreased decision conflict [30]	1
Decreased decision regret [30]	1
Decreased digestive disturbance [26]	1
Decreased pain [26]	1
Decreased readmissions^a^ [52]	1
Enabled preference for telemedicine [25]	1
Extended care to rural patients [28]	1
Increased medication adherence [41]	1
Reduced health costs per patient^a^ [27]	1
Administrative observations^a^	82

^a^Collected data that show effectiveness.

### Barriers to the Adoption of mHealth for Mental Health Care

Three barriers were identified in the literature for the adoption of mHealth for mental health care. mHealth and telemedicine may not be the preferred modality of treatment for some patients or providers. This, along with the requirement to train staff, was identified in the literature 33 times [25-57]. The other barrier was for countries that must receive reimbursement for care, and that telemedicine modalities are not often fully reimbursed to the point where they would pay for the intervention. This observation occurred 19 times [25-28,30,31,35-37,41-44,49-53,56].

### Interactions Between Interventions

When mHealth was used as the intervention, a reduction in both depression and anxiety was reported [26,27,29,30,32,33,35,36,38,39,41,43,45-50,53,55,56]. The same can be said for telemedicine [25,28,31,40,44,51,52], and mostly for telephone intervention [34,37,42,54]. Only 1 of each telephone [34] intervention and telemonitoring [57] intervention had no effect on depression or anxiety.

## Discussion

### Summary of Evidence

This systematic literature review analyzed 33 RCTs from 14 countries published over a 1-year period in peer-reviewed, academic journals using adults as participants (half of which were older adults) to analyze the effectiveness of mHealth for mental health care. A total of 4 interventions were studied: mHealth or eHealth apps, telemedicine (delivered over either a computer or a mobile device), telephone, and telemonitoring. Strong study methodologies resulted in low bias within and across studies. Observations of both sample and selection bias were noted, but there was nothing significant to report from these sources of bias. Overall, the interventions resulted in 26 instances of reduced depression; 19 instances of reduced anxiety; 7 instances of increased quality of life; 5 instances of increased mental well-being; 4 instances of decreased stress, increased self-efficacy, and increased sleep; 2 instances of decreased fatigue or increased resilience; and 1 instance each of decreased anger, decision regret, decision conflict, digestive disturbance, pain, readmission, and health care costs per patient. Only 2 studies reported no effect on depression and anxiety.

Future research should focus on standardizing mHealth into clinical practice guidelines for the treatment of some depression and anxiety issues. mHealth interventions can be rapidly deployed to a wide range of patient for very little money [27]. All but 2 studies reported improvements in at least one area of care [34,57]. This shows great promise for this modality of care.

Results from this systematic review should empower providers to adopt some mHealth interventions to augment or supplant existing practices; however, a few barriers should be addressed. While mHealth interventions can be conveniently adopted by some providers, it may not be the preferred modality for some patients. Providers should be sensitive to patient preferences. As mHealth and telemedicine modalities are introduced to provider clinics, staff training will have to take place, but after initial training has occurred, small refresher training should be all that is necessary. Finally, while many countries introduced reimbursement mechanisms during the pandemic, many have expired or have not been renewed. This is an important policy point that this review documents. It is imperative that the efficacy of this modality be recognized as beneficial to patients, and as such they should be reimbursed appropriately.

### Limitations

This study has several limitations. While confirmation bias can create problems for researchers, multiple reviewers were used to control for this bias. While selection bias can be a problem with internal validity, multiple research databases were used to control for this bias. Publication bias is one this study did not control for. Because we only used studies published in peer-reviewed academic journals, it is possible there are other studies without positive results that we failed to include in the analysis. Our review used a Boolean search string from MeSH to ensure the search was exhaustive, but this technique may have overlooked articles indexed with terms other than those in MeSH. The short time frame of acceptance criteria (1 year) may also have introduced a limitation because there may have been older studies with results worthy of analysis. Including these studies may have biased the results during the pandemic.

### Conclusions

mHealth is an effective tool to augment or, in some cases, supplant certain treatments of mental health care. It has been shown to improve depression and anxiety (primary research objectives) and many other conditions such as distress, sleep disturbance, pain, digestive disturbance, anger, fatigue, decision regret, and self-efficacy. Although some studies reported results that were not statistically significant, all but 2 interventions showed improvement in at least one area of care. These results are promising for both patients and providers seeking additional methods of care.

## Appendix

Multimedia Appendix 1 Observation-to-theme conversion (Intervention, Results, Medical Outcomes).

Multimedia Appendix 2 Observation-to-theme conversion (Patient Satisfaction, Effectiveness, Barriers).

Multimedia Appendix 3 Other observations incident to the data extraction process (sample size, country of origin, effect size, statistics used, JHNEBP strength and quality of evidence).

## Abbreviations

CINAHL: Cumulative Index to Nursing and Allied Health Literature
HADS: Hospital Anxiety and Depression Scale
HADS-A: Hospital Anxiety and Depression Scale-Anxiety
HADS-D: Hospital Anxiety and Depression Scale-Depression
JHNEBP: John’s Hopkins Nursing Evidence-Based Practice
MeSH: Medical Subject Headings
mHealth: mobile health
OR: odds ratio
PICOS: participants, intervention, comparison with the control or other group, medical outcomes, and study design
PTSD: posttraumatic stress disorder
RCT: randomized controlled trial
WoS: Web of Science

## Data Availability

Data from this study can be obtained by asking the lead author.

## References

[1] Freud S (2015). Civilization and its discontents.

[2] Gottman J (2017). The roles of conflict engagement, escalation, and avoidance in marital interaction: A longitudinal view of five types of couples. Interpersonal Development (1st Edition).

[3] Davison GC, Neale JM (1974). Abnormal Psychology: An Experimental Clinical Approach.

[4] Gilmore S (1973). The Counselor-in-Training.

[5] Muñoz R (1987). Depression Prevention: Research Directions.

[6] Turner BJ, Siegel S (2022). Telemedicine and mental health care of young adults during COVID-19: A qualitative study. Practice Innovations.

[7] Foa EB, Franklin M, McLean C, McNally RJ, Pine D, Jane Costello E, Franklin M, Kagan J, Kendall P, Klein R, Leonard H, Liebowitz M, March J, McNally R, Ollendick T, Pine D, Pynoos R, Silverman W, Spear L, Evans D (2017). Defining anxiety disorders. Treating and Preventing Adolescent Mental Health Disorders: What We Know and What We Don't Know (2nd Edition).

[8] Silva WAD, de Sampaio Brito TR, Pereira CR (2022). COVID-19 anxiety scale (CAS): Development and psychometric properties. Curr Psychol.

[9] World Health Organization (WHO) (2010). Telemedicine: Opportunities and Developments in Member States: Report on the Second Global Survey on eHealth.

[10] Kruse C, Heinemann K (2022). Facilitators and Barriers to the Adoption of Telemedicine During the First Year of COVID-19: Systematic Review. J Med Internet Res.

[11] Kruse CS, Beane A (2018). Health Information Technology Continues to Show Positive Effect on Medical Outcomes: Systematic Review. J Med Internet Res.

[12] World Health Organization (WHO) (2011). mHealth: New horizons for health through mobile technologies. WHO.

[13] Mitchell KM, Holtz BE, McCarroll AM (2022). Assessing College Students' Perceptions of and Intentions to Use a Mobile App for Mental Health. Telemed J E Health.

[14] Daly JR, Depp C, Graham SA, Jeste DV, Kim H, Lee EE, Nebeker C (2021). Health Impacts of the Stay-at-Home Order on Community-Dwelling Older Adults and How Technologies May Help: Focus Group Study. JMIR Aging.

[15] Massie MJ (2004). Prevalence of depression in patients with cancer. J Natl Cancer Inst Monogr.

[16] Park S (2022). Which of the Cornell Scale for Depression in Dementia or the Geriatric Depression Scale is more useful to screen for depression in older adults?. Asian J Psychiatr.

[17] Hu T, Zhao X, Wu M, Li Z, Luo L, Yang C, Yang F (2022). Prevalence of depression in older adults: A systematic review and meta-analysis. Psychiatry Res.

[18] Serrano-Ripoll MJ, Zamanillo-Campos R, Fiol-DeRoque MA, Castro A, Ricci-Cabello I (2022). Impact of Smartphone App-Based Psychological Interventions for Reducing Depressive Symptoms in People With Depression: Systematic Literature Review and Meta-analysis of Randomized Controlled Trials. JMIR Mhealth Uhealth.

[19] Noble D, Haveland S, Islam MS (2022). Integrating Telepsychiatry Based Care in Rural Acute Community Mental Health Services? A systematic literature review. Asia Pacific Journal of Health Management.

[20] Page M, McKenzie J, Bossuyt P, Boutron I, Hoffmann T, Mulrow C, Shamseer Larissa, Tetzlaff Jennifer M, Akl Elie A, Brennan Sue E, Chou Roger, Glanville Julie, Grimshaw Jeremy M, Hróbjartsson Asbjørn, Lalu Manoj M, Li Tianjing, Loder Elizabeth W, Mayo-Wilson Evan, McDonald Steve, McGuinness Luke A, Stewart Lesley A, Thomas James, Tricco Andrea C, Welch Vivian A, Whiting Penny, Moher David (2021). The PRISMA 2020 statement: an updated guideline for reporting systematic reviews. BMJ.

[21] Kruse CS (2019). Writing a Systematic Review for Publication in a Health-Related Degree Program. JMIR Res Protoc.

[22] Newhouse R, Dearholt S, Poe S, Pugh L, White K (2005). The Johns Hopkins nursing evidence-based practice rating scale. The Johns Hopkins Hospital.

[23] Pannucci CJ, Wilkins EG (2010). Identifying and avoiding bias in research. Plast Reconstr Surg.

[24] Braun V, Clarke V (2006). Using thematic analysis in psychology. Qualitative Research in Psychology.

[25] Acierno R, Jaffe AE, Gilmore AK, Birks A, Denier C, Muzzy W, Lopez CM, Tuerk P, Grubaugh AL (2021). A randomized clinical trial of in-person vs. home-based telemedicine delivery of Prolonged Exposure for PTSD in military sexual trauma survivors. J Anxiety Disord.

[26] Baek Y, Jeong K, Lee S, Kim H, Seo B, Jin H (2021). Feasibility and Effectiveness of Assessing Subhealth Using a Mobile Health Management App (MibyeongBogam) in Early Middle-Aged Koreans: Randomized Controlled Trial. JMIR Mhealth Uhealth.

[27] Colomina J, Drudis R, Torra M, Pallisó Francesc, Massip M, Vargiu E, Nadal N, Fuentes A, Ortega Bravo M, Miralles F, Barbé Ferran, Torres G, de Batlle J, CONNECARE-Lleida Group (2021). Implementing mHealth-Enabled Integrated Care for Complex Chronic Patients With Osteoarthritis Undergoing Primary Hip or Knee Arthroplasty: Prospective, Two-Arm, Parallel Trial. J Med Internet Res.

[28] Dobkin RD, Mann SL, Weintraub D, Rodriguez KM, Miller RB, St Hill L, King A, Gara MA, Interian A (2021). Innovating Parkinson's Care: A Randomized Controlled Trial of Telemedicine Depression Treatment. Mov Disord.

[29] Domogalla L, Beck A, Schulze-Hagen T, Herr R, Benecke J, Schmieder A (2021). Impact of an eHealth Smartphone App on the Mental Health of Patients With Psoriasis: Prospective Randomized Controlled Intervention Study. JMIR Mhealth Uhealth.

[30] Fang S, Lin P, Kuo Y (2021). Long-Term Effectiveness of a Decision Support App (Pink Journey) for Women Considering Breast Reconstruction Surgery: Pilot Randomized Controlled Trial. JMIR Mhealth Uhealth.

[31] Fortney JC, Bauer AM, Cerimele JM, Pyne JM, Pfeiffer P, Heagerty PJ, Hawrilenko M, Zielinski MJ, Kaysen D, Bowen DJ, Moore DL, Ferro L, Metzger K, Shushan S, Hafer E, Nolan JP, Dalack GW, Unützer Jürgen (2021). Comparison of Teleintegrated Care and Telereferral Care for Treating Complex Psychiatric Disorders in Primary Care: A Pragmatic Randomized Comparative Effectiveness Trial. JAMA Psychiatry.

[32] Huberty J, Puzia ME, Green J, Vlisides-Henry RD, Larkey L, Irwin MR, Vranceanu A (2021). A mindfulness meditation mobile app improves depression and anxiety in adults with sleep disturbance: Analysis from a randomized controlled trial. Gen Hosp Psychiatry.

[33] Jones RA, Mueller J, Sharp SJ, Vincent A, Duschinsky R, Griffin SJ, Ahern AL (2021). The impact of participant mental health on attendance and engagement in a trial of behavioural weight management programmes: secondary analysis of the WRAP randomised controlled trial. Int J Behav Nutr Phys Act.

[34] Krzyzanowska MK, Julian JA, Gu C, Powis M, Li Q, Enright K, Howell D, Earle CC, Gandhi S, Rask S, Brezden-Masley C, Dent S, Hajra L, Freeman O, Spadafora S, Hamm C, Califaretti N, Trudeau M, Levine MN, Amir E, Bordeleau L, Chiarotto JA, Elser C, Husain J, Laferriere N, Rahim Y, Robinson AG, Vandenberg T, Grunfeld E (2021). Remote, proactive, telephone based management of toxicity in outpatients during adjuvant or neoadjuvant chemotherapy for early stage breast cancer: pragmatic, cluster randomised trial. BMJ.

[35] Moskowitz JT, Addington EL, Shiu E, Bassett SM, Schuette S, Kwok I, Freedman ME, Leykin Y, Saslow LR, Cohn MA, Cheung EO (2021). Facilitator Contact, Discussion Boards, and Virtual Badges as Adherence Enhancements to a Web-Based, Self-guided, Positive Psychological Intervention for Depression: Randomized Controlled Trial. J Med Internet Res.

[36] Pakrad F, Ahmadi F, Grace SL, Oshvandi K, Kazemnejad A (2021). Traditional vs Extended Hybrid Cardiac Rehabilitation Based on the Continuous Care Model for Patients Who Have Undergone Coronary Artery Bypass Surgery in a Middle-Income Country: A Randomized Controlled Trial. Arch Phys Med Rehabil.

[37] Rollman BL, Anderson AM, Rothenberger SD, Abebe KZ, Ramani R, Muldoon MF, Jakicic JM, Herbeck Belnap B, Karp JF (2021). Efficacy of Blended Collaborative Care for Patients With Heart Failure and Comorbid Depression: A Randomized Clinical Trial. JAMA Intern Med.

[38] Romijn G, Batelaan N, Koning J, van Balkom A, de Leeuw A, Benning F, Hakkaart van Roijen L, Riper H (2021). Acceptability, effectiveness and cost-effectiveness of blended cognitive-behavioural therapy (bCBT) versus face-to-face CBT (ftfCBT) for anxiety disorders in specialised mental health care: A 15-week randomised controlled trial with 1-year follow-up. PLoS One.

[39] Su JJ, Yu DS (2021). Effects of a nurse-led eHealth cardiac rehabilitation programme on health outcomes of patients with coronary heart disease: A randomised controlled trial. Int J Nurs Stud.

[40] Taguchi K, Numata N, Takanashi R, Takemura R, Yoshida T, Kutsuzawa K, Yoshimura K, Nozaki-Taguchi N, Ohtori S, Shimizu E (2021). Clinical Effectiveness and Cost-effectiveness of Videoconference-Based Integrated Cognitive Behavioral Therapy for Chronic Pain: Randomized Controlled Trial. J Med Internet Res.

[41] Wong AKC, Wong FKY, Chow KKS, Wong SM, Lee PH (2021). Effect of a Telecare Case Management Program for Older Adults Who Are Homebound During the COVID-19 Pandemic: A Pilot Randomized Clinical Trial. JAMA Netw Open.

[42] Aikens JE, Valenstein M, Plegue MA, Sen A, Marinec N, Achtyes E, Piette JD (2022). Technology-Facilitated Depression Self-Management Linked with Lay Supporters and Primary Care Clinics: Randomized Controlled Trial in a Low-Income Sample. Telemed J E Health.

[43] Akin-Sari B, Inozu M, Haciomeroglu AB, Trak E, Tufan D, Doron G (2022). Cognitive training using a mobile app as a coping tool against COVID-19 distress: A crossover randomized controlled trial. J Affect Disord.

[44] Bathgate CJ, Kilbourn KM, Murphy NH, Wamboldt FS, Holm KE (2022). Pilot RCT of a telehealth intervention to reduce symptoms of depression and anxiety in adults with cystic fibrosis. J Cyst Fibros.

[45] Catuara-Solarz S, Skorulski B, Estella-Aguerri I, Avella-Garcia CB, Shepherd S, Stott E, Hemmings NR, Ruiz de Villa A, Schulze L, Dix S (2022). The Efficacy of "Foundations," a Digital Mental Health App to Improve Mental Well-being During COVID-19: Proof-of-Principle Randomized Controlled Trial. JMIR Mhealth Uhealth.

[46] Deady M, Glozier N, Calvo R, Johnston D, Mackinnon A, Milne D, Choi I, Gayed A, Peters D, Bryant R, Christensen H, Harvey SB (2022). Preventing depression using a smartphone app: a randomized controlled trial. Psychol Med.

[47] Drew RJ, Morgan PJ, Young MD (2022). Mechanisms of an eHealth program targeting depression in men with overweight or obesity: A randomised trial. J Affect Disord.

[48] Guo Y, Li Y, Yu C, Xu H, Hong YA, Wang X, Zhang N, Zeng Y, Monroe-Wise A, Li L, Liu C, Cai W, Lin A (2022). Long-term Effects of a Social Media-Based Intervention (Run4Love) on Depressive Symptoms of People Living With HIV: 3-Year Follow-up of a Randomized Controlled Trial. J Med Internet Res.

[49] Gustafson DH, Kornfield R, Mares M, Johnston DC, Cody OJ, Yang EF, Gustafson DH, Hwang J, Mahoney JE, Curtin JJ, Tahk A, Shah DV (2022). Effect of an eHealth intervention on older adults' quality of life and health-related outcomes: a randomized clinical trial. J Gen Intern Med.

[50] Kuhn E, Miller KE, Puran D, Wielgosz J, YorkWilliams SL, Owen JE, Jaworski BK, Hallenbeck HW, McCaslin SE, Taylor KL (2022). A Pilot Randomized Controlled Trial of the Insomnia Coach Mobile App to Assess Its Feasibility, Acceptability, and Potential Efficacy. Behav Ther.

[51] Lopez CM, Gilmore AK, Brown WJ, Hahn CK, Muzzy W, Grubaugh A, Acierno R (2022). Effects of Emotion Dysregulation on Post-treatment Post-traumatic Stress Disorder and Depressive Symptoms Among Women Veterans With Military Sexual Trauma. J Interpers Violence.

[52] Mitchell SE, Reichert M, Howard JM, Krizman K, Bragg A, Huffaker M, Parker K, Cawley M, Roberts HW, Sung Y, Brown J, Culpepper L, Cabral HJ, Jack BW (2022). Reducing Readmission of Hospitalized Patients With Depressive Symptoms: A Randomized Trial. Ann Fam Med.

[53] Nardi W, Roy A, Dunsiger S, Brewer J (2022). Analyzing the Impact of Mobile App Engagement on Mental Health Outcomes: Secondary Analysis of the Unwinding Anxiety Program. J Med Internet Res.

[54] Orman Z, Thrift AG, Olaiya MT, Ung D, Cadilhac DA, Phan T, Nelson MR, Srikanth VK, Vuong J, Bladin CF, Gerraty RP, Fitzgerald SM, Frayne J, Kim J, STANDFIRM (Shared Team Approach between NursesDoctors For Improved Risk factor Management) Investigators (2022). Quality of life after stroke: a longitudinal analysis of a cluster randomized trial. Qual Life Res.

[55] Sun S, Lin D, Goldberg S, Shen Z, Chen P, Qiao S, Brewer J, Loucks E, Operario D (2022). A mindfulness-based mobile health (mHealth) intervention among psychologically distressed university students in quarantine during the COVID-19 pandemic: A randomized controlled trial. J Couns Psychol.

[56] Volpato E, Banfi P, Pagnini F (2022). Promoting Acceptance and Adherence to Noninvasive Ventilation in Chronic Obstructive Pulmonary Disease: A Randomized Controlled Trial. Psychosom Med.

[57] Ware P, Shah A, Ross HJ, Logan AG, Segal P, Cafazzo JA, Szacun-Shimizu K, Resnick M, Vattaparambil T, Seto E (2022). Challenges of Telemonitoring Programs for Complex Chronic Conditions: Randomized Controlled Trial With an Embedded Qualitative Study. J Med Internet Res.

