# Supplementary material for: Leveraging Mobile Health to Manage Mental Health/Behavioral Health Disorders: Systematic Literature Review
Source: JMIR Ment Health. 2022 Dec 27;9(12):e42301. doi: 10.2196/42301 (PMC9832355; doi:10.2196/42301)
Supplement: Multimedia Appendix 1 [file mental_v9i12e42301_app1.docx]

**Appendix A:** Observation-to-theme conversion (Intervention, Results, Medical Outcomes).

| Authors | Experimental Intervention | Intervention themes | Results (compared to control group) | Results Themes | Medical Outcomes Reported | Medical Outcomes Themes |  |
| --- | --- | --- | --- | --- | --- | --- | --- |
| Acierno et al [25] | Telemedicine | Telemedicine | Reduced depression, but there were no differences in dose received or PTSD symptom reduction | Reduced depression | Decrease in depression, but not statistically significant | Reduced depression |  |
|  |  |  |  | No statistical significance for at least one condition |  |  |  |
|  |  |  |  |  |  |  |  |
| Baek et al [26] | mHealth app (MibyeongBogam, MBBG) | mHealth / eHealth app | Intervention group showed decrease in depression (*p*=.003), anxiety (*p*=.01), sleep disturbance (*p*=.02), anger (*p*=.003), pain (*p*=.02) greater than the control -- Also fatigue (*p*=.6), digestive disturbance (*p*=.76) not statistically significant | Reduced depression | Decreased depression, anxiety, sleep disturbance, anger, pain, fatigue, digestive disturbance | Reduced depression |  |
|  |  |  |  | Reduced anxiety |  | Reduced anxiety |  |
|  |  |  |  | Increased sleep |  | Increased sleep |  |
|  |  |  |  | Decreased anger |  | Decreased anger |  |
|  |  |  |  | Decreased pain |  | Decreased pain |  |
|  |  |  |  | Decreased digestive disturbance |  | Decreased digestive disturbance |  |
|  |  |  |  | No statistical significance for at least one condition |  |  |  |
| Colomina et al [27] | mHealth self-management app (CONNECARE) | mHealth / eHealth app | Decreased anxiety more than the control group, but there were no differences in depression symptom reduction | Reduced anxiety | Decreased anxiety, but not statistically significant over traditional care | Reduced anxiety |  |
|  |  |  |  | No effect on depression |  |  |  |
|  |  |  |  | No statistical significance for at least one condition |  |  |  |
| Dobkin et al [28] | video-to-home cognitive-behavioral therapy | Telemedicine | Intervention outperformed treatment as usual across all 3 measures of depression (*p*=.001), decreased anxiety, but not statistically significant. | Reduced depression | Decreased depression and anxiety | Reduced anxiety |  |
|  |  |  |  | Reduced anxiety |  | Reduced depression |  |
|  |  |  |  | No statistical significance for at least one condition |  |  |  |
| Domogalla et al [29] | mHealth study and disease management app | mHealth / eHealth app | Significant reduction in hospital anxiety and depression scale (HADS), HADS-D (*p*=.04) and HADS-A (*p*=.05) more than the control group | Reduced anxiety | Decreased anxiety and depression | Reduced anxiety |  |
|  |  |  |  | Reduced depression |  | Reduced depression |  |
|  |  |  |  |  |  |  |  |
| Fang et al [30] | mHealth app (Pink Journey) | mHealth / eHealth app | Decreased anxiety, depression, decision conflict, and decision regret more than the control, but not statistically significant from control, decreased body-image distress (*p*=.027) | Reduced anxiety | Decreased anxiety, depression, decision conflict, decision regret, and body image distress | Reduced anxiety |  |
|  |  |  |  | Reduced depression |  | Reduced depression |  |
|  |  |  |  | Decreased decision conflict |  | Decreased decision conflict |  |
|  |  |  |  | Decreased decision regret |  | Decreased decision regret |  |
|  |  |  |  | Decreased distress |  | Decreased distress |  |
|  |  |  |  | No statistical significance for at least one condition |  |  |  |
| Fortney et al [31] | telepsychiatry | Telemedicine | Decreased depression & anxiety, but with small effect | Reduced anxiety | Decreased depression and anxiety | Reduced anxiety |  |
|  |  |  |  | Reduced depression |  | Reduced depression |  |
| Huberty et al [32] | mHealth app | mHealth / eHealth app | Decreased anxiety (*p*<.001) and depression (*p*<.001) more than the control group | Reduced anxiety | Decreased anxiety and depression | Reduced anxiety |  |
|  |  |  |  | Reduced depression |  | Reduced depression |  |
| Jones et al [33] | mHealth app (WRAP) | mHealth / eHealth app | Decreased HADS more than the control group | Reduced anxiety | Decreased anxiety and depression | Reduced anxiety |  |
|  |  |  |  | Reduced depression |  | Reduced depression |  |
| Kryzanowska et al [34] | telephone | Telephone | No effect on anxiety, depression, or self-efficacy | No effect on anxiety | No effect on anxiety, depression, or self-efficacy | None |  |
|  |  |  |  | No effect on depression |  |  |  |
|  |  |  |  | No effect on self-efficacy |  |  |  |
| Moskowitz et al [35] | eHealth | mHealth / eHealth app | Decreased depression (*p*<.06) more than the control group | Reduced depression | Decreased depression | Reduced depression |  |
| Pakrad et al [36] | mHealth app | mHealth / eHealth app | Decreased anxiety (*p*<.028), stress (*p*<.022), and quality of life (*p*<.001) more than the control. Decreased depression more than control, but not statistically significant (*p*<.063). | Reduced anxiety | Decreased depression, anxiety, and stress, and increased quality of life | Reduced anxiety |  |
|  |  |  |  | Decreased distress |  | Decreased distress |  |
|  |  |  |  | Increased quality of life |  | Increased quality of life |  |
|  |  |  |  | Reduced depression |  | Reduced depression |  |
|  |  |  |  | No statistical significance for at least one condition |  |  |  |
| Rollman et al [37] | Telephone | Telephone | Decreased depression more than the control group | Reduced depression | Decreased depression | Reduced depression |  |
| Romijn et al [38] | eHealth cognitive behavior therapy (iCBT) | mHealth / eHealth app | Decreased anxiety more than the control group | Reduced anxiety | Decreased anxiety | Reduced anxiety |  |
| Su & Yu [39] | eHealth | mHealth / eHealth app | Decreased anxiety more than the control group, no effect on depression | Reduced anxiety | Decreased anxiety | Reduced anxiety |  |
|  |  |  |  | No effect on depression |  |  |  |
| Taguchi et a [40]l | Video-based CBT | Telemedicine | Decreased depression and anxiety (not statistically significant from control) | Reduced anxiety | Decreased depression and anxiety | Reduced anxiety |  |
|  |  |  |  | Reduced depression |  | Reduced depression |  |
|  |  |  |  | No statistical significance for at least one condition |  |  |  |
| Wong et al [41] | mHealth app | mHealth / eHealth app | Decreased depression (not statistically significant over control), increased medication adherence (*p*<.001), self-efficacy (*p*<.16), and quality of life (*p*<.04) | Reduced depression | Decreased depression, improved medication adherence, self-efficacy, and quality of life | Reduced depression |  |
|  |  |  |  | No statistical significance for at least one condition |  |  |  |
|  |  |  |  | Increased medication adherence |  | Increased medication adherence |  |
|  |  |  |  | Increased self-efficacy |  | Increased self-efficacy |  |
|  |  |  |  | Increased quality of life |  | Increased quality of life |  |
| Aikens et al [42] | Telephone (automated interactive voice response, IVR) | Telephone | Decreased depression more than the control group, with medium effect. Increased self-efficacy | Reduced depression | Decreased depression, increased self-efficacy | Reduced depression |  |
|  |  |  |  | Increased self-efficacy |  | Increased self-efficacy |  |
| Akin-Sari et al [43] | mHealth app | mHealth / eHealth app | Decreased depression and COVID-19 distress more than the control group | Reduced depression | Decreased depression, decreased COVID-19 distress | Reduced depression |  |
|  |  |  |  | Decreased distress |  | Decreased distress |  |
| Bathgate et al [44] | telemedicine | Telemedicine | Decreased depression more than control (*p*=.78), anxiety but not more than the control (*p*=.6), increased coping self-efficacy but not more than control (*p*=.93), increased QOL (physical functioning, social functioning, and vitality) | Reduced depression | Decreased depression & anxiety, increased coping self-efficacy and QOL | Reduced depression |  |
|  |  |  |  | Reduced anxiety |  | Reduced anxiety |  |
|  |  |  |  | Increased self-efficacy |  | Increased self-efficacy |  |
|  |  |  |  | Increased quality of life |  | Increased quality of life |  |
|  |  |  |  | No statistical significance for at least one condition |  |  |  |
| Catuara-Solarz et al [45] | mHealth app | mHealth / eHealth app | Decreased anxiety (P=.04), increase in resilience (*p*=.001), sleep (*p*=.01), and mental well-being (*p*=.02) more than the control group | Reduced anxiety | Decreased anxiety, increase in resilience, sleep, and mental well-being | Reduced anxiety |  |
|  |  |  |  | Decreased fatigue / increased resilience |  | Decreased fatigue / increased resilience |  |
|  |  |  |  | Increased sleep |  | Increased sleep |  |
|  |  |  |  | Increased mental well-being / cognitive flexibility |  | Increased mental well-being / cognitive flexibility |  |
| Deady et al [46] | mHealth app (HeadGear) | mHealth / eHealth app | Improved depression, anxiety, resilience, and well-being more than the control group (*p*=.0031) | Reduced depression | Improved depression, anxiety, resilience, and well-being | Reduced depression |  |
|  |  |  |  | Reduced anxiety |  | Reduced anxiety |  |
|  |  |  |  | Decreased fatigue / increased resilience |  | Decreased fatigue / increased resilience |  |
|  |  |  |  | Increased mental well-being / cognitive flexibility |  | Increased mental well-being / cognitive flexibility |  |
| Drew et al [47] | eHealth app (SHED-IT) | mHealth / eHealth app | Improved depression, sleep, cognitive flexibility more than the control | Reduced depression | Improved depression, sleep, cognitive flexibility | Reduced depression |  |
|  |  |  |  | Increased sleep |  | Increased sleep |  |
|  |  |  |  | Increased mental well-being / cognitive flexibility |  | Increased mental well-being / cognitive flexibility |  |
| Guo et al [48] | mHealth, social-media (Run4Love) | mHealth / eHealth app | Improved depression more than control | Reduced depression | Decreased depression | Reduced depression |  |
| Gustafson et al [49] | eHealth app (ElderTree) | mHealth / eHealth app | Improved depression (OR=-0.20, *p*=.034)and overall mental health quality of life (OR=0.32, *p*=.007) more than the control group | Reduced depression | Decreased depression, increased mental health, increased quality of life | Reduced depression |  |
|  |  |  |  | Increased mental well-being / cognitive flexibility |  | Increased mental well-being / cognitive flexibility |  |
|  |  |  |  | Increased quality of life |  | Increased quality of life |  |
| Kuhn et al [50] | mHealth app | mHealth / eHealth app | Decreased depression (*d*=-0.8, *p*<.012) and sleep-related impairment (*d*=-0.6, *p*<.04) more than the control group | Reduced depression | Decreased depression and sleep related impairment | Reduced depression |  |
|  |  |  |  | Increased sleep |  | Increased sleep |  |
| Lopez et al [51] | Telemedicine | Telemedicine | Reduced depression, but there were no differences in dose received or PTSD symptom reduction | Reduced depression | Decrease in depression, but not statistically significant | Reduced depression |  |
|  |  |  |  | No statistical significance for at least one condition |  |  |  |
| Mitchell et al [52] | Telemedicine CBT (RED-D) | Telemedicine | Decreased depression and readmission (*p*<.012) more than the control | Reduced depression | Decreased depression | Reduced depression |  |
| Nardi et al [53] | mHealth app (unwinding anxiety) | mHealth / eHealth app | Decreased anxiety (*p*=.005) and worry (*p*=.01) more than the control | Reduced anxiety | Decreased anxiety and worry | Reduced anxiety |  |
|  |  |  |  | Decreased distress |  | Decreased distress |  |
| Orman et al [54] | Telephone | Telephone | Decreased depression and anxiety greater than usual care, short-term positive effect on quality of life | Reduced depression | Decreased anxiety and depression, and increased quality of life | Reduced depression |  |
|  |  |  |  | Reduced anxiety |  | Reduced anxiety |  |
|  |  |  |  | Increased quality of life |  | Increased quality of life |  |
| Sun et al [55] | mHealth app (mindfulness) | mHealth / eHealth app | Decreased depression and anxiety (*p*=.024) greater than usual care, but depression was not statistically different | Reduced depression | Decreased anxiety and depression | Reduced depression |  |
|  |  |  |  | Reduced anxiety |  | Reduced anxiety |  |
|  |  |  |  | No statistical significance for at least one condition |  |  |  |
| Volpato et al [56] | mHealth CBT | mHealth / eHealth app | Decreased anxiety and depression, but not statistically significant over the control. Improved adherence to noninvasive ventilation (*p*<.001) and quality of life (*p*<.002) | Reduced anxiety | Decreased anxiety and depression, improved quality of life and noninvasive ventilation | Reduced anxiety |  |
|  |  |  |  | Reduced depression |  | Reduced depression |  |
|  |  |  |  | Increased quality of life |  | Increased quality of life |  |
| Ware et al [57] | Telemonitoring | Telemonitoring | No effect on anxiety or depression. Improved self-care maintenance, management, confidence, and physical quality of life. | No effect on anxiety | Improved self-care maintenance, management, confidence, and physical quality of life. | No effect on anxiety |  |
|  |  |  |  | No effect on depression |  | No effect on depression |  |
|  |  |  |  | Increased self-efficacy |  | Increased self-efficacy |  |
|  |  |  |  | Increased mental well-being / cognitive flexibility |  | Increased mental well-being / cognitive flexibility |  |
|  |  |  |  | Increased quality of life |  | Increased quality of life |  |
