# Supplementary material for: Leveraging Mobile Health to Manage Mental Health/Behavioral Health Disorders: Systematic Literature Review
Source: JMIR Ment Health. 2022 Dec 27;9(12):e42301. doi: 10.2196/42301 (PMC9832355; doi:10.2196/42301)
Supplement: Multimedia Appendix 2 [file mental_v9i12e42301_app2.docx]

**Appendix B:** Observation-to-theme conversion (Patient Satisfaction, Effectiveness, Barriers).

| Authors | Patient Satisfaction | Patient Satisfaction Themes | Effectiveness | Effectiveness Themes | Barriers to adoption | Barrier Themes |  |
| --- | --- | --- | --- | --- | --- | --- | --- |
| Acierno et al [25] | Greater satisfaction than traditional care (not statistically significant) | Satisfied | Decreases depression, enabling preferences for HBT | Reduced depression | May not be preferable, staff training, low reimbursement | May not be preferred treatment method |  |
|  |  |  |  | Enabled preference for telemedicine |  | Staff training |  |
|  |  |  |  |  |  | Low reimbursement |  |
| Baek et al [26] | Not reported | Not reported | Decreased depression, anxiety, sleep disturbance, anger, pain, fatigue, digestive disturbance | Reduced depression | May not be preferable, staff training, low reimbursement | May not be preferred treatment method |  |
|  |  |  |  | Reduced anxiety |  | Staff training |  |
|  |  |  |  | Increased sleep |  | Low reimbursement |  |
|  |  |  |  | Decreased anger |  |  |  |
|  |  |  |  | Decreased pain |  |  |  |
|  |  |  |  | Decreased digestive disturbance |  |  |  |
|  |  |  |  |  |  |  |  |
| Colomina et al [27] | User experience was satisfactory | Satisfied | Reduce health costs per patient, reduced number of visits to facility, decreased anxiety | Reduced health costs per patient | May not be preferable, staff training, low reimbursement | May not be preferred treatment method |  |
|  |  |  |  | Reduced anxiety |  | Staff training |  |
|  |  |  |  |  |  | Low reimbursement |  |
| Dobkin et al [28] | High satisfaction | Satisfied | Decreased depression and anxiety, extended reach of care | Reduced anxiety | May not be preferable, staff training, low reimbursement | May not be preferred treatment method |  |
|  |  |  |  | Reduced depression |  | Staff training |  |
|  |  |  |  | Extended care to rural patients |  | Low reimbursement |  |
| Domogalla et al [29] | High satisfaction | Satisfied | Decreased anxiety and depression | Reduced anxiety | May not be preferable, staff training | May not be preferred treatment method |  |
|  |  |  |  | Reduced depression |  | Staff training |  |
| Fang et al [30] | High satisfaction | Satisfied | Decreased anxiety, depression, decision conflict, decision regret, and body image distress | Reduced anxiety | May not be preferable, staff training, low reimbursement | May not be preferred treatment method |  |
|  |  |  |  | Reduced depression |  | Staff training |  |
|  |  |  |  | Decreased decision conflict |  | Low reimbursement |  |
|  |  |  |  | Decreased decision regret |  |  |  |
|  |  |  |  | Decreased distress |  |  |  |
| Fortney et al [31] | Not reported | Not reported | Decreased depression and anxiety | Reduced anxiety | May not be preferable, staff training, low reimbursement | May not be preferred treatment method |  |
|  |  |  |  | Reduced depression |  | Staff training |  |
|  |  |  |  |  |  | Low reimbursement |  |
| Huberty et al [32] | High satisfaction | Satisfied | Decreased depression and anxiety | Reduced anxiety | May not be preferable, staff training, low reimbursement | May not be preferred treatment method |  |
|  |  |  |  | Reduced depression |  | Staff training |  |
| Jones et al [33] | High satisfaction | Satisfied | Decreased depression and anxiety | Reduced anxiety | May not be preferable, staff training | May not be preferred treatment method |  |
|  |  |  |  | Reduced depression |  | Staff training |  |
| Kryzanowska et al [34] | Not reported | Not reported | No effect on anxiety, depression, or self-efficacy | None | May not be preferable, staff training | May not be preferred treatment method |  |
|  |  |  |  |  |  | Staff training |  |
| Moskowitz et al [35] | High satisfaction | Satisfied | Decreased depression | Reduced depression | May not be preferable, staff training, low reimbursement | May not be preferred treatment method |  |
|  |  |  |  |  |  | Staff training |  |
|  |  |  |  |  |  | Low reimbursement |  |
| Pakrad et al [36] | positive perception of quality of care | Satisfied | Decreased depression, anxiety, and stress, and increased quality of life | Reduced anxiety | May not be preferable, staff training, low reimbursement | May not be preferred treatment method |  |
|  |  |  |  | Decreased distress |  | Staff training |  |
|  |  |  |  | Reduced depression |  | Low reimbursement |  |
|  |  |  |  | Increased quality of life |  |  |  |
| Rollman et al [37] | Not reported | Not reported | Decreased depression | Reduced depression | May not be preferable, staff training, low reimbursement | May not be preferred treatment method |  |
|  |  |  |  |  |  | Staff training |  |
|  |  |  |  |  |  | Low reimbursement |  |
| Romijn et al [38] | High satisfaction | Satisfied | Decreased anxiety | Reduced anxiety | May not be preferable, staff training | May not be preferred treatment method |  |
|  |  |  |  |  |  | Staff training |  |
| Su & Yu [39] | Not reported | Not reported | Decreased anxiety | Reduced anxiety | May not be preferable, staff training | May not be preferred treatment method |  |
|  |  |  |  |  |  | Staff training |  |
| Taguchi et al [40] | Not reported | Not reported | Decreased depression and anxiety | Reduced anxiety | May not be preferable, staff training | May not be preferred treatment method |  |
|  |  |  |  | Reduced depression |  | Staff training |  |
|  |  |  |  |  |  |  |  |
| Wong et al [41] | Not reported | Not reported | Decreased depression, improved medication adherence, self-efficacy, and quality of life | Reduced depression | May not be preferable, staff training, low reimbursement | May not be preferred treatment method |  |
|  |  |  |  |  |  | Staff training |  |
|  |  |  |  | Increased medication adherence |  | Low reimbursement |  |
|  |  |  |  | Increased self-efficacy |  |  |  |
|  |  |  |  | Increased quality of life |  |  |  |
| Aikens et al [42] | Not reported | Not reported | Decreased depression, increased self-efficacy | Reduced depression | May not be preferable, staff training, low reimbursement | May not be preferred treatment method |  |
|  |  |  |  | Increased self-efficacy |  | Staff training |  |
|  |  |  |  |  |  | Low reimbursement |  |
| Akin-Sari et al [43] | Not reported | Not reported | Decreased depression, decreased COVID-19 distress | Reduced depression | May not be preferable, staff training, low reimbursement | May not be preferred treatment method |  |
|  |  |  |  | Decreased distress |  | Staff training |  |
|  |  |  |  |  |  | Low reimbursement |  |
| Bathgate et al [44] | High satisfaction | Satisfied | Decreased depression & anxiety, increased coping self-efficacy and QOL | Reduced depression | May not be preferable, staff training, low reimbursement | May not be preferred treatment method |  |
|  |  |  |  | Reduced anxiety |  | Staff training |  |
|  |  |  |  | Increased self-efficacy |  | Low reimbursement |  |
|  |  |  |  | Increased quality of life |  |  |  |
| Catuara-Solarz et al [45] | High satisfaction | Satisfied | Decreased anxiety, increase in resilience, sleep, and mental well-being | Reduced anxiety | May not be preferable, staff training | May not be preferred treatment method |  |
|  |  |  |  | Decreased fatigue / increased resilience |  | Staff training |  |
|  |  |  |  | Increased sleep |  |  |  |
|  |  |  |  | Increased mental well-being / cognitive flexibility |  |  |  |
| Deady et al [46] | Not reported | Not reported | Improved depression, anxiety, resilience, and well-being | Reduced depression | May not be preferable, staff training | May not be preferred treatment method |  |
|  |  |  |  | Reduced anxiety |  | Staff training |  |
|  |  |  |  | Decreased fatigue / increased resilience |  |  |  |
|  |  |  |  | Increased mental well-being / cognitive flexibility |  |  |  |
| Drew et al [47] | Not reported | Not reported | Improved depression, sleep, cognitive flexibility | Reduced depression | May not be preferable, staff training | May not be preferred treatment method |  |
|  |  |  |  | Increased sleep |  | Staff training |  |
|  |  |  |  | Increased mental well-being / cognitive flexibility |  |  |  |
| Guo et al [48] | Not reported | Not reported | Decreased depression | Reduced depression | May not be preferable, staff training | May not be preferred treatment method |  |
|  |  |  |  |  |  | Staff training |  |
| Gustafson et al [49] | Not reported | Not reported | Decreased depression, increased mental health, increased quality of life | Reduced depression | May not be preferable, staff training, low reimbursement | May not be preferred treatment method |  |
|  |  |  |  | Increased mental well-being / cognitive flexibility |  | Staff training |  |
|  |  |  |  | Increased quality of life |  | Low reimbursement |  |
| Kuhn et al [50] | Not reported | Not reported | Decreased depression and sleep related impairment | Reduced depression | May not be preferable, staff training, low reimbursement | May not be preferred treatment method |  |
|  |  |  |  | Increased sleep |  | Staff training |  |
|  |  |  |  |  |  | Low reimbursement |  |
| Lopez et al [51] | Not reported | Not reported | Decreased depression | Reduced depression | May not be preferable, staff training, low reimbursement | May not be preferred treatment method |  |
|  |  |  |  |  |  | Staff training |  |
|  |  |  |  |  |  | Low reimbursement |  |
| Mitchell et al [52] | High satisfaction | Satisfied | Decreased depression and readmission | Reduced depression | May not be preferable, staff training, low reimbursement | May not be preferred treatment method |  |
|  |  |  |  | Decreased readmissions |  | Staff training |  |
|  |  |  |  |  |  | Low reimbursement |  |
| Nardi et al [53] | Not reported | Not reported | Decreased anxiety and worry | Reduced anxiety | May not be preferable, staff training, low reimbursement | May not be preferred treatment method |  |
|  |  |  |  | Decreased distress |  | Staff training |  |
|  |  |  |  |  |  | Low reimbursement |  |
| Orman et al [54] | Not reported | Not reported | Decreased anxiety and depression, and a short-term positive effect on quality of life | Reduced depression | May not be preferable, staff training | May not be preferred treatment method |  |
|  |  |  |  | Reduced anxiety |  | Staff training |  |
|  |  |  |  | Increased quality of life |  |  |  |
| Sun et al [55] | Not reported | Not reported | Decreased anxiety and depression | Reduced depression | May not be preferable, staff training | May not be preferred treatment method |  |
|  |  |  |  | Reduced anxiety |  | Staff training |  |
|  |  |  |  |  |  |  |  |
| Volpato et al [56] | Not reported | Not reported | Decreased anxiety and depression, improved quality of life and noninvasive ventilation | Reduced anxiety | May not be preferable, staff training, low reimbursement | May not be preferred treatment method |  |
|  |  |  |  | Reduced depression |  | Staff training |  |
|  |  |  |  | Increased quality of life |  | Low reimbursement |  |
| Ware et al [57] | Not reported | Not reported | Improved self-care maintenance, management, confidence, and physical quality of life. | Increased self-efficacy | May not be preferable, staff training | May not be preferred treatment method |  |
|  |  |  |  | Increased mental well-being / cognitive flexibility |  | Staff training |  |
|  |  |  |  | Increased quality of life |  |  |  |
