# Supplementary material for: Leveraging Mobile Health to Manage Mental Health/Behavioral Health Disorders: Systematic Literature Review
Source: JMIR Ment Health. 2022 Dec 27;9(12):e42301. doi: 10.2196/42301 (PMC9832355; doi:10.2196/42301)
Supplement: Multimedia Appendix 3 [file mental_v9i12e42301_app3.docx]

**Appendix C:** Other observations incident to the data extraction process (sample size, country of origin, effect size, statistics used, JHNEBP strength and quality of evidence).

| Authors | Sample Size | Bias within study | Effect Size | Country of Origin (where was the study conducted?) | Statistics Used | Strength of Evidence | Quality of Evidence |  |
| --- | --- | --- | --- | --- | --- | --- | --- | --- |
| Acierno et al [25] | 136 | Multiple sites across one large country, female only and 64% African American (sample bias) | Not reported | US | Descriptive statistics, Wald tests, | I | A |  |
|  |  |  |  |  |  |  |  |  |
| Baek et al [26] | 150 | One country (selection bias), 67% female, 100% Korean (sample bias) | Not reported | Korea | Descriptive statistics, *t*-tests, Chi-square tests, ANCOVA | I | A |  |
| Colomina et al [27] | 59 | One country (selection bias), 66% female (sample bias) | Not reported | Spain | Descriptive statistics, measures of central tendency, binomial regression, *t*-tests | I | A |  |
| Dobkin et al [28] | 90 | Multiple sites across one large country, male only and 83% Caucasian (sample bias) | HAMD Large (1.27), BDI large (0.80), HAMA large (1.17), BAS medium (0.61), ATQ medium (0.60), PCS medium (0.62) | US | Descriptive statistics, Linear Mixed Models, Fisher's exact test, ANCOVA | I | A |  |
| Domogalla et al [29] | 77 | One region of one country (selection bias), 60% male (sample bias) | Not reported | Germany | Descriptive statistics, Linear Panel Data Regression | I | A |  |
| Fang et al [30] | 80 | One country (selection bias), 100% female (sample bias) | Not reported | Taiwan | Descriptive statistics, *t*-tests, Chi-square tests | I | A |  |
| Fortney et al [31] | 1004 | One country (selection bias), 70% female 66% Caucasian (sample bias) | Small effect (*d*=0.15) | US | Descriptive statistics, Mixed Models, Cohen's *d* | I | A |  |
| Huberty et al [32] | 239 | One country (selection bias), 78% female 56% Caucasian (sample bias) | medium effect (0.50) | US | Descriptive statistics, *t*-tests, Chi-square tests, ANCOVA | I | A |  |
| Jones et al [33] | 1267 | One country (selection bias) | Small effect | UK | Descriptive statistics, logistic regression, Chi-square test, odds ratio, binomial regression | I | A |  |
| Kryzanowska et al [34] | 561 | Multiple sites in one country | Not reported | Canada | Descriptive statistics, Poisson model (negative binomial distribution), mixed effects logistic model | I | A |  |
| Moskowitz et al [35] | 602 | One country (selection bias), 74% female (sample bias) | small (*w*=0.2) | US | Descriptive statistics, Chi-square | I | A |  |
| Pakrad et al [36] | 88 | One country (selection bias), 82% male (sample bias) | small (0.115) | Iran | Descriptive statistics, Chi-square, paired *t*-tests, Bonferroni adjustments | I | A |  |
| Rollman et al [37] | 756 | One country (selection bias), high % one race (sample bias) | medium (0.47) | US | Descriptive statistics, Chi-square | I | A |  |
| Romijn et al [38] | 114 | One country (selection bias), high % of one race (sample bias) | small (*d*=-0.38) | Netherlands | Descriptive statistics, *t*-tests, linear mixed model with restricted maximum likelihood, Bonferroni-Hold correction, generalized linear model, Cohen *d* | I | A |  |
| Su & Yu [39] | 146 | One country (selection bias) high % male and one ethnicity (sample bias) | medium (0.45) | China | Descriptive statistics, *t*-tests, Chi-square test, generalized estimating equation model, Bonferroni correction | I | A |  |
| Taguchi et al [40] | 30 | One country (selection bias), high % female (sample bias) | sensitivity 75%, specificity 90% | Japan | Descriptive statistics, *t*-tests, ANCOVA | I | A |  |
| Wong et al [41] | 68 | One country (selection bias), high % female (sample bias) | Sensitivity 89%, specificity 77% | Hong Kong | Descriptive statistics, 2-sided *t*-tests, Mann-Whitney *U* | I | A |  |
| Aikens et al [42] | 204 | One country (selection bias), high % female and race (sample bias) | medium (0.38) | US | Descriptive statistics, logistic regression, 2-tailed *t*-tests | I | A |  |
| Akin-Sari et al [43] | 40 | One country (selection bias), high % female (sample bias) | large effect (*d*=0.81 - 2.35), Odds ratio 2.2 (twice as likely to reduce depression) | Turkey | Descriptive statistics, MANOVA, Pearson Product moment correlation, *t*-tests, Bonferroni correction | I | A |  |
| Bathgate et al [44] | 31 | One country (selection bias), high % female and race (sample bias) | large (*d*=0.74 - 0.95) | US | Descriptive statistics, *t*-tests, Chi-square tests, Cohen's *d* | I | A |  |
| Catuara-Solarz et al [45] | 136 | One country (selection bias) | Not reported | Spain | Descriptive statistics, 2-tailed *t*-tests, Bonferroni correction, linear mixed models for between-group analysis | I | A |  |
| Deady et al [46] | 2271 | One country (selection bias), high % male (sample bias) | small | UK | Descriptive statistics, 2-sided t-tests, ANOVA | I | A |  |
| Drew et al [47] | 125 | One country (selection bias), all male (sample bias) | Not reported | Australia | Descriptive statistics, Chi-square | I | A |  |
| Guo et al [48] | 300 | One country (selection bias), high % male and one ethnicity (sample bias) | medium (*d*=0.39) | China | Descriptive statistics, Chi-square, generalized estimating equation, linear regression, Cohen *d* | I | A |  |
| Gustafson et al [49] | 310 | One country (selection bias), high % female and one ethnicity (sample bias) | medium (*d*=0.4) | US | Descriptive statistics, cumulative link mixed models | I | A |  |
| Kuhn et al [50] | 50 | One country (selection bias), mostly male (sample bias) | large effect (*d*=0.8) | US | Descriptive statistics, Rigorous and Accelerated Data Reduction (RaDaR), Linear mixed model, Fisher's exact test, *t*-tests | I | A |  |
| Lopez et al [51] | 151 | One country (selection bias), high % female (sample bias) | Not reported | US | Descriptive statistics, linear regression | I | A |  |
| Mitchell et al [52] | 709 | One country (selection bias), high % female (sample bias) | Not reported | US | Descriptive statistics, incidence rate ratios, Poisson models | I | A |  |
| Nardi et al [53] | 27 | One country (selection bias), high % female (sample bias) | Not reported | US | Descriptive statistics, Spearman rank correlation, multivariate linear regression | I | B |  |
|  |  |  |  |  |  |  |  |  |
| Orman et al [54] | 563 | One country (selection bias), high % male (sample bias) | Not reported | Australia | Descriptive statistics, multivariable, mixed-effects linear regression | I | A |  |
| Sun et al [55] | 99 | One country (selection bias), high % one ethnicity (sample bias) | anxiety large effect (*d*=0.72), depression medium effect (*d*=0.36) | China | Descriptive statistics, Chi-squared | I | A |  |
| Volpato et al [56] | 61 | One country (selection bias) | Not reported | US | Descriptive statistics, mixed model analysis | I | A |  |
| Ware et al [57] | 54 | One country (selection bias) | Not reported | Canada | Descriptive statistics | I | A |  |
